# Editorial

**Published:** 1872-09-15

**Authors:** 


### Medical Examiner.

*A Semi-Monthly Journal of Medical Sciences.*

EDITED BY

N. S. DAVIS, M. D., AND F. H. DAVIS, M. D.

Chicago, September 15th, 1872.

### EDITORIAL.

Malarious Influence.—We hear from almost all directions that intermittent and remittent fevers are more prevalent this season than usual. Not only is this true in reference to the greater part of Illinois, Indiana, Michigan, and Iowa, but also of the southern part of Wisconsin.

A short time since we were informed by an active practitioner in Janesville, that these fevers were more prevalent in some parts of that city than for seventeen years previously.

This unusual prevalence of ague is not limited to the Western States, however. In New York and Brooklyn, and their suburbs, we are told the same unusual prevalence of malarial diseases exists.

In some of the southern townships of this (Cook) county, sickness of this character has been so prevalent as to leave hardly well persons enough to take care of the sick. In this city we have had but little pure and simple intermittent fever, but almost all cases have presented a mixed or typho-malarial character. The same influence has doubtless added much both to the prevalence and mortality of the bowel complaints of children. The leading cause of this wide-spread and unusual prevalence of malaria is supposed to be the small amount of *rainfall* during the present and past summer, and the consequent greater evaporation from the sub-soil.

Credit to whom Credit is due.—A brief report on the prevalence of cerebro-spinal disease in Chicago, which was read to the Chicago Medical Society, and originally published in the Medical Examiner, is copied into the September number of the *Journal of Materia Medica*, and credited to the *Kansas City Medical Journal* for June, 1872.

The subjoined obituary is translated from *El Nacional*, a paper in Quito, South America, and commemorates the death of Dr. Casares, Professor of Surgery in the University of Quito. Professor Casares was the author of one of the letters on Cundurango published in the Examiner. He was a man of immense energy, and commanded unbounded admiration and esteem in the city of Quito.

E. Andrews, M. D.

